# Ranavirus genotypes in the Netherlands and their potential association with virulence in water frogs (*Pelophylax* spp.)

**DOI:** 10.1038/s41426-018-0058-5

**Published:** 2018-04-04

**Authors:** Bernardo Saucedo, Joseph Hughes, Annemarieke Spitzen-van der Sluijs, Natasja Kruithof, Marc Schills, Jolianne M. Rijks, Mónica Jacinto-Maldonado, Nicolás Suarez, Olga L. M. Haenen, Michal Voorbergen-Laarman, Jan van den Broek, Maarten Gilbert, Andrea Gröne, Steven J. van Beurden, M. Hélène Verheije

**Affiliations:** 1 Department of Pathobiology, Faculty of Veterinary Medicine, Utrecht University, Utrecht, 3584 CL The Netherlands; 2 MRC-University of Glasgow Centre for Virus Research, Glasgow, G61 1QH UK; 3 Reptile, Amphibian and Fish Conservation the Netherlands (RAVON), Nijmegen, 6525 ED The Netherlands; 4 Dutch Wildlife Health Centre (DWHC), Utrecht, 3584 CL The Netherlands; 5 Department of Etology, Wildlife and Laboratory Animals, Faculty of Veterinary Medicine, Autonomous University of Mexico (UNAM), Mexico city, 04510 Mexico; 6 Wageningen Bioveterinary Research (WBVR) of Wageningen UR, Lelystad, 8200 AB The Netherlands; 7 Department of Farm Animal Health, Faculty of Veterinary Medicine, Utrecht University, Utrecht, 3584 CL The Netherlands; 8 Present Address: Gupta Strategists, Ophemert, 4060 GA The Netherlands

## Abstract

Ranaviruses are pathogenic viruses for poikilothermic vertebrates worldwide. The identification of a common midwife toad virus (CMTV) associated with massive die-offs in water frogs (*Pelophylax* spp.) in the Netherlands has increased awareness for emerging viruses in amphibians in the country. Complete genome sequencing of 13 ranavirus isolates collected from ten different sites in the period 2011–2016 revealed three CMTV groups present in distinct geographical areas in the Netherlands. Phylogenetic analysis showed that emerging viruses from the northern part of the Netherlands belonged to CMTV-NL group I. Group II and III viruses were derived from the animals located in the center-east and south of the country, and shared a more recent common ancestor to CMTV-amphibian associated ranaviruses reported in China, Italy, Denmark, and Switzerland. Field monitoring revealed differences in water frog host abundance at sites where distinct ranavirus groups occur; with ranavirus-associated deaths, host counts decreasing progressively, and few juveniles found in the north where CMTV-NL group I occurs but not in the south with CMTV-NL group III. Investigation of tandem repeats of coding genes gave no conclusive information about phylo-geographical clustering, while genetic analysis of the genomes revealed truncations in 17 genes across CMTV-NL groups II and III compared to group I. Further studies are needed to elucidate the contribution of these genes as well as environmental variables to explain the observed differences in host abundance.

## Introduction

Ranaviruses (family *Iridoviridae*) are double-stranded DNA viruses, known to cause disease in fish, reptiles, and amphibians^1–3^ and are notifiable to the World Organization for Animal Health (OIE)^4^. They are the second most common infectious cause of mortality in amphibians worldwide, after the fungus *Batrachochytrium dendrobatidis* (*Bd*)^1, 5^. The ranavirus common midwife toad virus (CMTV) was first reported in Spain and identified as the cause of a die-off in 2007 which involved juvenile alpine newts (*Ichthyosaura alpestris cyreni*) and larval stages of the common midwife toad (*Alytes obstetricans*)^6^. Later work showed that die-offs had been occurring at the site since 2005^7^. Shortly thereafter, a CMTV-related mass die-off affecting water frogs (*Pelophylax* spp.) and smooth newts (*Lissotriton vulgaris*) took place in the northern part of the Netherlands in 2010^8, 9^. The oldest records of CMTV ranaviruses are from 1995 and correspond to isolates from pike perch in Finland^10^ and common frogs in Great Britain^11^.

In subsequent years, CMTV ranaviruses continued to be detected in multiple other countries, affecting wild or captive water frogs in Denmark^12^, Italy^12, 13^, and Switzerland^14^, captive colonies of Hermann’s tortoises (*Testudo hermanni*) in Switzerland^3, 15^, wild common frogs (*Rana temporaria*) in the French Alps^16^, wild Lake Urmia newts (*Neurergus crocatus*) from Iraq^17^, and captive Chinese giant salamanders (*Andrias davidianus*) in China^18^. It is not clear whether CMTV was already present in these countries but had previously gone undetected, or if these outbreaks were the result of recent introductions via trade^19^, migratory birds^20^, or invertebrates^21^.

Since the first report of CMTV in the Netherlands in 2010, the presence of ranavirus in the Dutch amphibian population has been monitored by the Dutch Wildlife Health Centre (DWHC) and Reptile, Amphibian and Fish Conservation in the Netherlands (RAVON). Based on partial genome sequencing of viruses detected in the period 2010–2013, at least two distinct CMTV groups appeared to be present in the Netherlands: CMTV-NL Group I was identified as an emerging virus causing an on-going epidemic with high mortality rates in the north, while a distinct CMTV-NL group was associated with sporadic events of low mortality in the south^22^.

The aims of this study were to investigate the phylogenetic relationship of Dutch ranaviruses with other fully sequenced ranaviruses from around the globe, investigate whether there is a correlation between viral genotype and their effect in affected host populations in the field and in vitro, analyze factors in the field that influence ranavirus infections in susceptible water frogs, and to study dispersal and short-term evolution of Dutch ranaviruses from the emerging CMTV-NL group I. Hereto, the genomes of different CMTV-NL isolates were sequenced and phylogenetically-classified, the in vitro growth rates were characterized, and host abundance was monitored during outbreaks.

## Results

### Three distinct CMTV groups circulate in different areas of the Netherlands

**Fig. 1:**
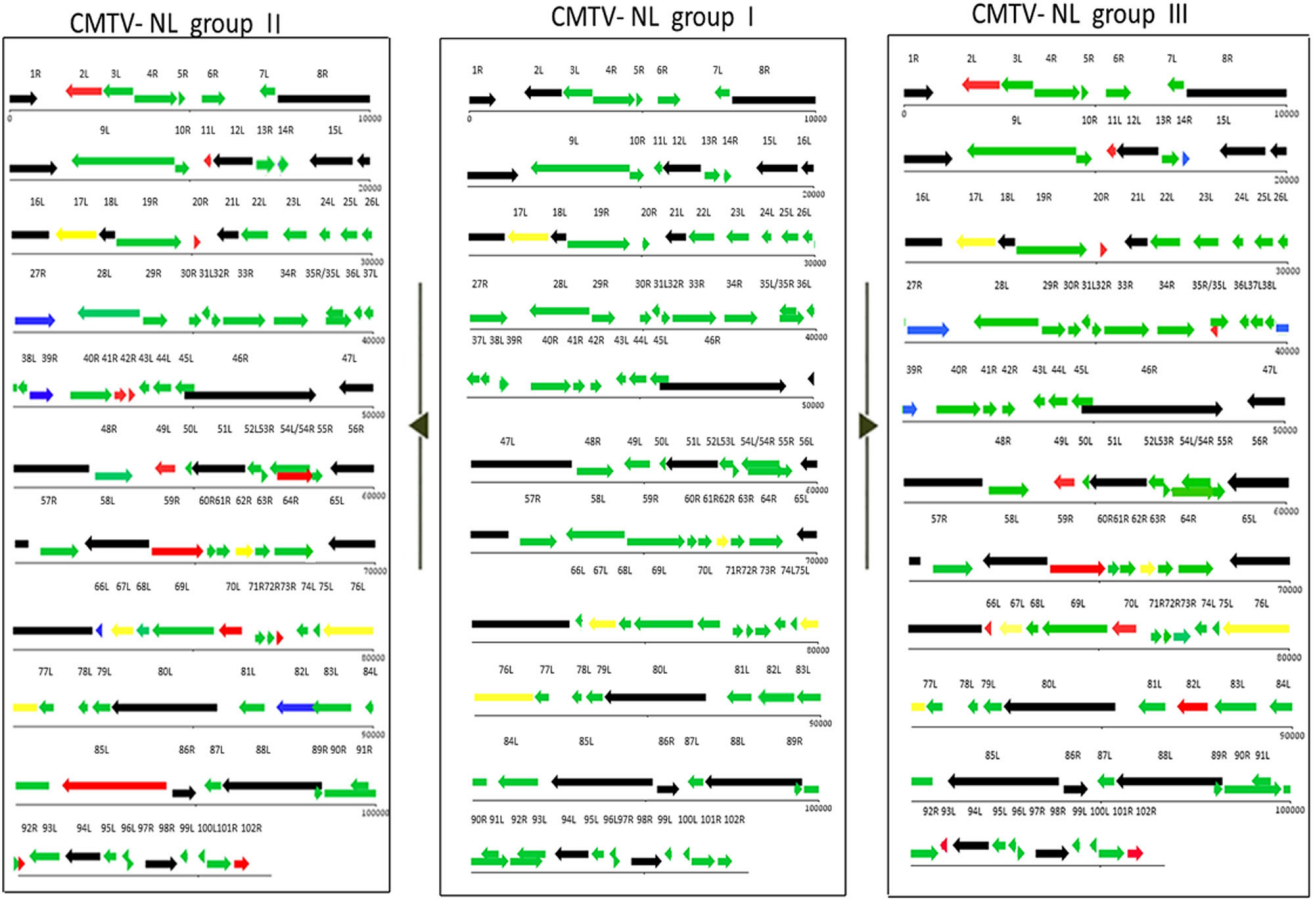
Genome layouts of three main CMTV-NL groups. A representative layout of group I viruses is featured in the middle section to facilitate comparison to CMTV NL II (left) and III (right). Red arrows represent shortened ORFs with the white portion of the arrows representing the amount of lost nucleotides/amino acids. Blue arrows represent genes with ORFs larger than their counterparts in CMTV-NL group I. Black arrows represent core iridoviral proteins. Green arrows represent genes identical in all groups

**Fig. 2:**
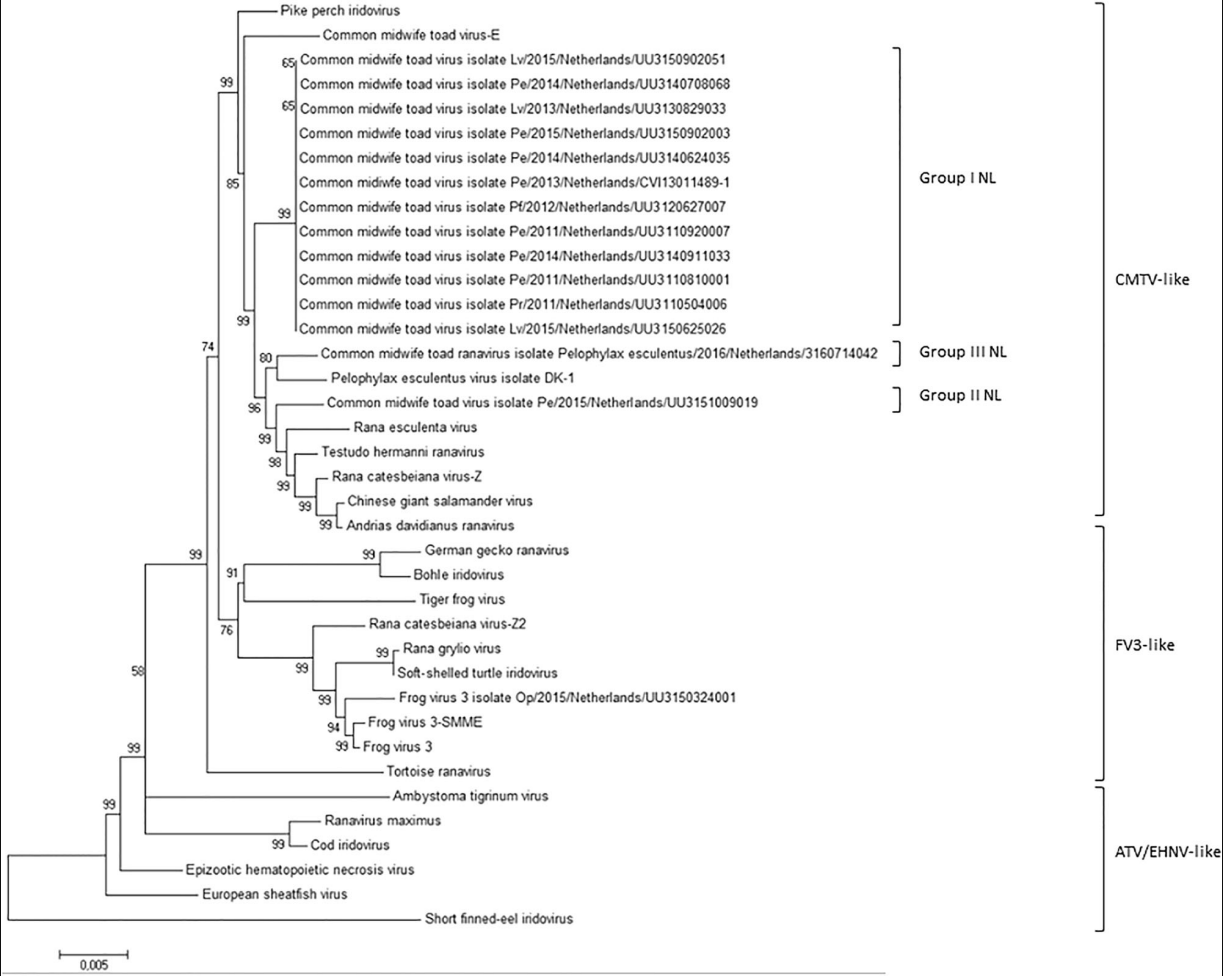
Ranavirus phylogenetic tree based on 50 genes (1000 bootstrap values). All Dutch ranaviruses cluster within the CMTV-like clade. Group II and III cluster closely with CMTV-like ranaviruses from China (ADRV, CGSIV), Italy (REV), Switzerland (THR), and Denmark (PEV-DEK1). Isolates and Genbank numbers used: common midwife toad ranavirus isolate *Pelophylax* kl.*esculentus*/2013/NL (KP056312), common midwife toad ranavirus isolate *Mesotriton alpestris*/2008/E (JQ231222), *Rana esculenta* virus isolate REV 282/I02 (MF538628), *Pelophylax esculentus* virus isolate PEV_DK1 (MF538627), *Rana catesbeiana* virus 1 isolate RCV-Z (MF187210), *R. catesbeiana* virus 2 isolate RCV2-Z2 (MF187209), *Frog virus 3* (AY548484), *Frog virus 3* isolate SSME (KJ175144), tortoise ranavirus isolate 1 (882/96) (KP266743), *Testudo hermanni* ranavirus isolate CH8/96 (KP266741), German gecko ranavirus isolate 2000/99 (KP266742), tiger frog virus (AF389451), soft shelled turtle iridovirus (EU627010), European sheathfish virus (JQ724856), *Epizootic hematopoietic necrosis virus* (FJ433873), *Ambystoma tigrinum stebbensi* virus (AY150217), *Andrias davidianus* ranavirus isolate 1201 (KC865735), Chinese giant salamander iridovirus, isolate CGSIV-HN1104, (KF512820), *Rana grylio* ranavirus (JQ654586), pike perch iridovirus isolate SLU 144001 (KX574341), *Bohle iridovirus* isolate BIV-ME 93/95, cod iridovirus isolate GAM14001 (KX57432), ranavirus maximus isolate SMA15001 (N_C030842), and short-finned eel ranavirus isolate ANGA14001 (KX353311)

Complete sequencing of 13 ranavirus isolates showed the presence of 3 distinct CMTV-NL groups in the Netherlands. A full genome layout of three Dutch ranaviruses belonging to the three main groups is shown in Fig. 1. CMTV-NL group I ranaviruses form a monophyletic clade together with the previously described isolate CMTV-*Pelophylax* kl. *esculentus*/2013/NL:^9^ All Dutch ranavirus isolates belonging to this group shared an overall sequence similarity of over 99.00%. CMTV group II, found in the east, showed 97.55% sequence similarity to CMTV-NL group I and 99.95% nucleotide similarity (total of 2367 nucleotides) with partial sequences of a ranavirus previously detected in the south in 2013 (Genbank no. KT003499)^22^. CMTV-NL group III from the south had 97.67% sequence similarity with CMTV-NL group I. CMTV-NL group II was shown to form a sister clade along with CMTV-like viruses from China (Chinese giant salamander virus and *A. davidianus* ranavirus)^23^ and *T. hermanni* ranavirus from Switzerland^3, 15^, while CMTV-NL group III was most closely related to a ranavirus from Denmark^12^. The phylogeny of all Dutch ranaviruses is shown in Fig. 2.

### Gene truncations found in groups II and III in comparison to group I

Individual genes of CMTV-NL groups I, II, and III were compared to study genetic differences between the groups. All three CMTV-NL groups were 100% identical in 25 out of the 26 genes conserved among the family *Iridoviridae*^24^. In addition, 16 other genes were found to show truncations in both CMTV-NL groups II and III compared to CMTV-NL group I (Table 1). Among these, only six are known to have a predicted function, while the others are hypothetical proteins. A few of these genes (2L, 20R, and 102R) were previously shown to be subject to positive selection^25^.

**Table 1:** Comparison of individual genes between CMTV-NL groups I, II, and III

Gene	Predicted function	NL-I	NL-II	NL-II Mutations/Locations	NL-III	NL-III Mutations/locations
2L	Myristolated membrane protein	361 aa	330 aa	c.1706_1744del, c.1719_177del, c.1853_1867del	325 aa	c.1706_1744del, c.1719_177del, c.1853_1867del
11L	Hypothetical protein	84 aa	66 aa	c.15506_15507delGA, 15593delA	76 aa	c.15593delA
20R	Hypothetical protein	71 aa	61 aa	c.25253_25282del	61 aa	c.25253_25282del
35R	Hypothetical protein	238 aa	238 aa	NA	65 aa	c.39165 G > T Stop codon
41R	Hypothetical protein	120 aa	117 aa	c.43380delC	120 aa	NA
42R	Hypothetical protein	114 aa	32 aa	32	114 aa	NA
49L	Hypothetical protein	248 aa	184 aa	c.55171delT	184 aa	c.55171delT
54L	Hypothetical protein	369 aa	369 aa	NA	229 aa	c.58251 G > A
59R	Hypothetical protein	562 aa	478 aa	c.64875_64922del,c.65051_65052del	487 aa	c.64091–64931del
66L	Hypothetical protein	46 aa	54 aa	54	46 aa	NA
70L	Putative NIF/NLI interacting factor	211 aa	209 aa	c.76602_76607del	211 aa	NA
73R	Hypothetical protein	155 aa	66 aa	c.78357_78350delCC premature stop codon	155 aa	NA
82L	p31K	305 aa	403 aa	c.89023_89024dupA	262 aa	c.89033dupA
85L	Putative D5 family NTPase/ATPase	975 aa	965 aa	c.94968_94997del	975 aa	NA
92R	Hypothetical protein	315 aa	27 aa	c.100970dupC premature stop codon	311 aa	c.101099_101110del
93L	Putative integrase like protein	275 aa	275 aa	NA	17 aa	c.101925delT premature stop codon
102R	Myeloid cell leukemia protein	145 aa	141 aa	c.107277_107289del	141 aa	c.107277_107289del

*NA* Not applicable as no mutations resulting in a decreased number of aminoacids occurred in comparison to CMTV-NL group I

### Mutations and variation in repetitive regions show no phylo-geographical clustering

No correlation could be found between the distance of CMTV-NL group I isolates to the index site (Supplementary Figure S1) and the number of mutations undergone in 5 years (Supplementary Table S1).

Out of the 102 putative genes annotated, six were shown to be variable in all viruses: the hypothetical protein genes 17L, 33R, 64R, 67L, and neurofilament triplet-H1 like protein genes 62R and 76L. Of these, 17L, 62R, and 67L were rich in tandem repeats and thus analyzed to check whether this number of tandem repeats would allow for differentiation of closely related strains or would coincide with specific geographical clustering as suggested previously^26–28^.

The number of repeats in gene 17L ranged between 7 and 13 for group I viruses (Table 2). Less variation was observed in the number of repeats between isolates in this gene than in the other genes studied. The number of tandem repeats observed for gene 62R was highly variable. It ranged from 4 to 19 repeats among the 13 fully sequenced virus isolates and the 20 additional virus sequences. Repeats were variable regardless of the site of origin, host species, or virus group. Variation of sequence nucleotide composition also differed slightly between group I and group III (Table 2). Regarding gene 67L, the analysis of the fully sequenced viruses revealed once again high inter-group variation (4–11 repeats). In this gene, the number of repeats in groups II and III were lower (2–4 repeats) than those of group I ranaviruses (6–10 repeats).

**Table 2:** Analysis of ranavirus repetitive regions

Gene	Sequence	Isolate no.	Year of collection	NL-strain	No. repeats for fully sequenced viruses	No. repeats for additional virus sequences from same site
17L	AGCAACGCCCTGCTCAGCAGC	1	2011	I	7	NA
		2	2011	I	10	NA
		3	2011	I	11	NA
		4a^a^, 4b^b^	2012, 2015	I	13 (4a^a^,4b^b^)	11 (2/5^a^), 13 (3/5^a^)
		5^b^	2014	I	11	NA
		6a, 6b	2013, 2014	I	7 (6a, 6b)	NA
		7a, 7b	2014, 2015	I	10 (7a, 7b)	10 (2/2; 3/3^b^)
		8	2015	I	7	7 (5/5)
		9	2015	II	7	NA
		10	2016	III	7	7 (5/5)
62R	AAGAGATCACCAGTGAAG	1	2011	I	17	NA
		2	2011	I	15	NA
		3	2011	I	13	NA
		4a^a^, 4b^b^	2012, 2015	I	9 (4a^a^), 5 (4b^b^)	8 (1/5), 7 (1/5), 6 (3/5)
		5^b^	2014	I	11	NA
		6a, 6b	2013, 2014	I	6 (6a), 13 (6b)	NA
		7a, 7b	2014, 2015	I	6 (7a), 14 (7b)	19 (1/5), 15 (3/5), 14 (1/5)
		8	2015	I	12	12 (5/5)
		9	2015	II	6	NA
	AAGAGCTCACCCGTGAAG	10	2016	III	4	8 (4/5), 10 (1/5)
67L	CCACTCAGAGTCCTACCA	1	2011	I	10	NA
		2	2011	I	9	NA
		3	2011	I	9	NA
		4a^a^, 4b^b^	2012, 2015	I	7 (4a^a^), 6 (4b^b^)	13 (1/5), 10 (2/5), 7 (1/5), 6 (1/5)
		5^b^	2014	I	8	NA
		6a, 6b	2013, 2014	I	9 (6a,6b)	NA
		7a, 7b	2014, 2015	I	11 (7a,7b)	11 (3/5),10 (1/5), 9 (1/5)
		8	2015	I	9	9 (5/5)
		9	2015	II	4	NA
		10	2016	III	2	2 (5/5)

^a^ *Pelobates fuscus*

^b^ *Lissotriton vulgaris*

Other species were all *Pelophylax*

### In vitro virus replication is similar between CMTV-NL groups

**Fig. 3:**
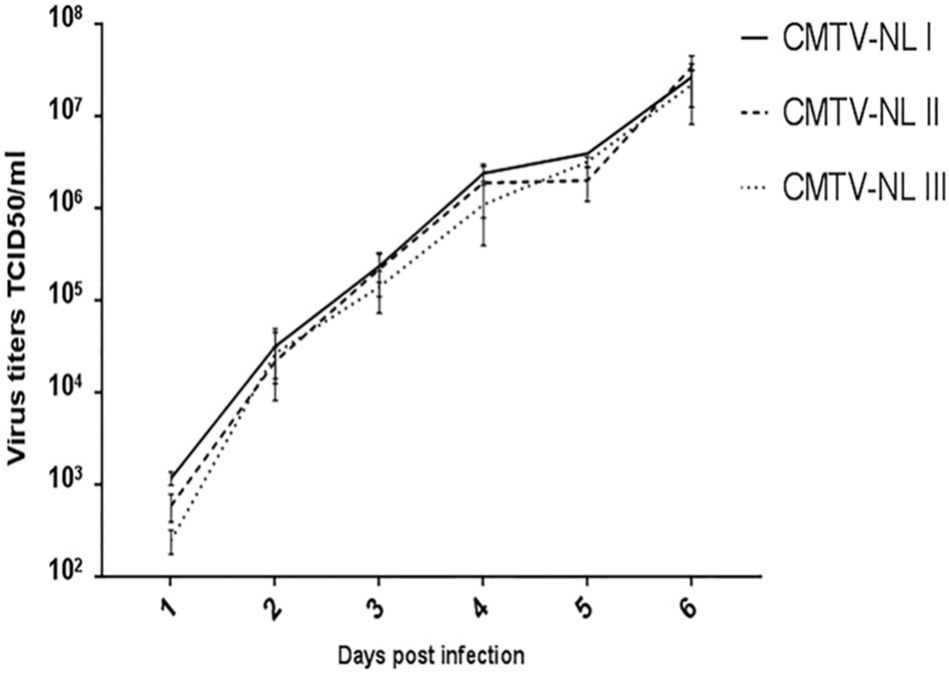
Multi-step growth curve assay. The figure depicts the average titer of each virus group and time point from three independent experiments. Error bars represent standard deviation

All viruses replicated with the same kinetics and reached comparable virus titers of approximately 1 × 10^7^ TCID_50_/ml at 6 days post infection (Fig. 3). This indicates that all three virus groups had similar in vitro growth characteristics. Consistent with the results of the growth curve assay, the unpaired *t*-test did not reveal any significant differences among the mean titer values of the three viruses. (CMTV-NL I vs. CMTVNL II (−12817715.23 to 16785311.35; tailed *p* value = 0.90), (CMTV-NL I vs. CMTV-NL III (−11316841.05 to 1360980.29; tailed *p* value = 0.84), (CMTV-NLII vs. CMTV-NL III (−16569803.52 to 148895246.65; tailed *p* value = 0.77).

### Water frog abundance differs between sites with distinct ranavirus groups

**Fig. 4:**
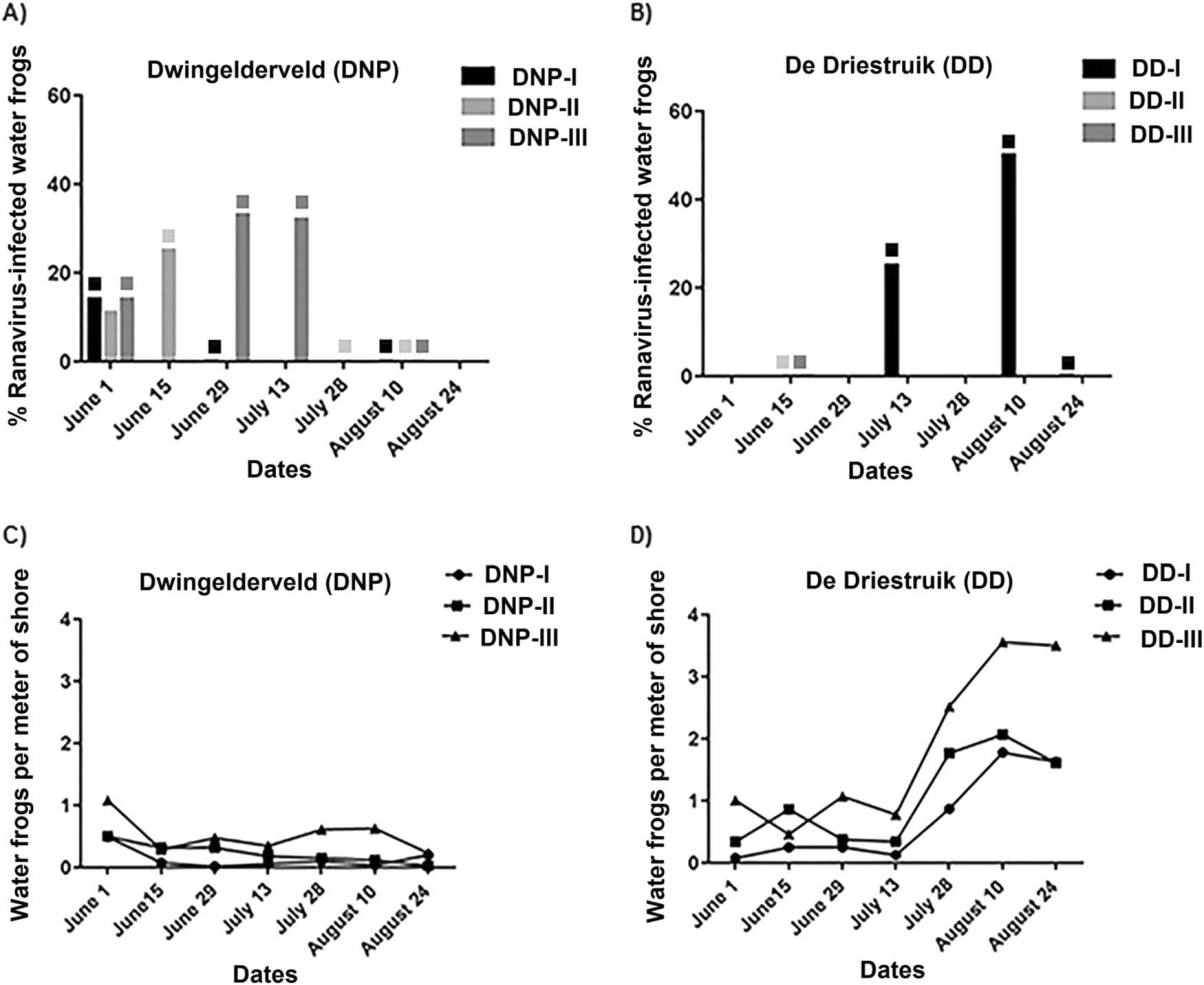
Proportion of ranavirus in swabbed specimens and adult water frog abundance (**a**, **b**) Ranavirus was detected from swabs and percentage of positive animals at each site is given. Water samples positive for ranavirus are indicated by blocks matching the color of the waterbody; (**c**, **d**) The ratio of water frog per shore meter is depicted for each visit for all waterbodies. No water frogs could be found for swabbing at DNP-I in visits June 15, June 29, July 13, July 28, and August 24, or in DNP-II from visit July 28 onwards

Ranaviral DNA was detected in water samples from all six waterbodies, and on swabbed or dead specimens in four water bodies (Fig. 4). Among the swabs from live amphibians, ranavirus DNA was detected primarily in swabs from water frogs (DNP:11/102; DD:10/225). In addition, at DNP the swabs of one smooth newt (DNP 1/15; DD:0/53) and of one live crested newt (DNP: 1/19; DD:0/0) tested positive. Other swabbed amphibian species tested negative for ranavirus presence by PCR (Table S2A). Partial characterization on six positive swab samples using seven genes as described previously^7, 22^, confirmed the groups were distributed as expected (DNP: group I; DD: group III). The endogenous extraction control (chloroplast mitochondria gene) was positive in all water samples (data not shown). The pH in all waterbodies from DNP and DD was 6.0, while water temperatures ranged from 14.0 to 22.3 °C in DNP (Supplementary table S2A) and 17.5 to 29.3 °C in DD (Supplementary Table S2B).

The number of water frogs per meter of shoreline tended to be lower in the waterbodies where ranavirus was detected on swabbed specimens (ranging from 0.02 to 0.49 frogs/m for DNP-I, 0.03 to 0.50 for DNP-II, 0.23 to 1.08 for DNP-III, and 0.09 to 1.79 for DD-I) compared to waterbodies where ranaviral DNA was detected in water only, ranging from 1.00 to 6.09 for DD-II and 0.46 to 3.51 for DD-III (Supplementary Table S2A and S2B). Ranaviral DNA was detected in all waterbodies in DNP from the beginning of the monitoring and then detected intermittently during subsequent visits (Supplementary Table S2A). The finding of ranavirus-positive water samples always coincided with finding positive swabs or confirmed dead specimens with ranaviral disease, except the last two visits at DNP-I, when counts of water frogs per meter of shoreline were extremely low (0.02 and 0.04 m). At DD, ranaviral DNA was detected in all waterbodies in at least one of the visits, but ranavirus positive animals were only detected at DD-I.

A seeming decrease in post-metamorphic water frog sightings per meter of shoreline was observed at DNP (DNP-I on June 15th and DNP-III on August 24th), accounting for a decrease in counts of around 80% in comparison to the first visit on June 1st. Water frog numbers increased slightly (40%) at DNP-I by the last visit in August 24th (Fig. 4c). In contrast, at DD despite evidence of ranavirus-positive water in all sites and a proportion of ranavirus-positive swabs that reached up to 50% (9/18) on August 10th, the number of (sub-) adult sightings per meter of shoreline was more than three-fold higher at all three sites by the end of the study (Fig. 4d). The specimens caught at DNP-I and DNP-II were mainly adult and subadult water frogs with juveniles very rarely caught, In contrast, at all ponds in DD and at DNP-III, juveniles were frequently caught from the period of July 28th to August 24th. Information on life stages and lengths of animals caught is available in Supplementary Table S3.

**Fig. 5:**
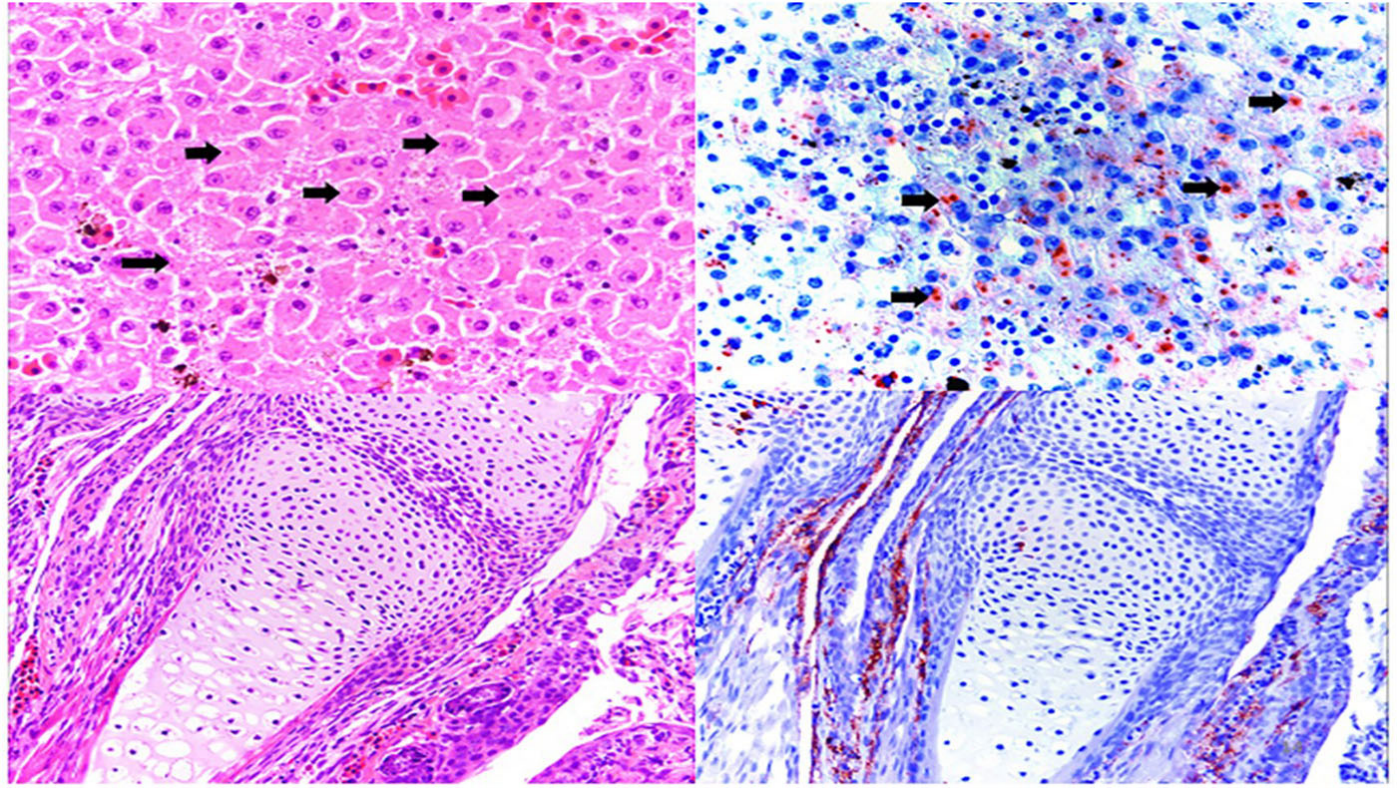
Histopathology and immunohistochemistry of ranavirus-infected water frogs. Top panel: (left) Hematoxylin/Eosin staining of a liver section from an affected water frog adult from DNP with intracytoplasmic basophilic inclusions (black arrows) and (right) immunohistochemical staining of a serial section in which intracytoplasmic inclusions present with marked immunolabelling confirming active viral replication. Lower panel: (left) hematoxylin/eosin staining of forelimb from affected water frog larva with no apparent microscopic lesion; (right) immunohistochemical staining of a serial section, in which positive immunolabelling is present in the periarticular muscle and connective tissue

Water frogs at DNP experienced ranavirus-associated mortality with a total of four adult specimens and three larvae, found at three different dates (Supplementary Table S2A). Four of the dead animals from DNP were examined microscopically and all showed classical ranavirus-associated lesions, such as presence of intracytoplasmic inclusion bodies and necrosis in various organs as well as positive immunolabelling for ranavirus antigen in tissues (Fig. 5). In contrast at DD, there was no evidence for adult water frog mortality with only a single dead ranavirus-positive larva displaying hemorrhages and edema during the second to last visit (August 10th) at DD-I. Pond DD-II showed a mortality event involving seven water frog and smooth newt larvae out of which only two could be collected due to adverse weather. However, PCR and immunohistochemistry for ranavirus were negative in both animals.

The fungus *Bd* was detected in amphibian swabs from all waterbodies except from DNP-I (DNP: 5/165; DD: 20/284) (data not shown). All dead animals tested negative for *Bd* by PCR and no compatible lesions were found during histopathological assessment.

In summary, at DNP where CMTV-NL group I is found, the water repeatedly tested positive and the number of water frog sightings decreased. In addition, there was evidence of ranavirus-infected specimens in all waterbodies, and ranavirus-associated deaths occurred on multiple occasions in both larvae and adults. In contrast, in DD where CMTV-NL group III is found, ranaviral DNA was detected in water of two of the waterbodies but no infected animals were found. The water of the third waterbody tested intermittently positive for ranavirus and had live-infected animals, but ranavirus–associated mortality only occurred in a single water frog larva.

### Mean frog counts per meter of shore correlates positively with group of ranavirus

The backward stepwise Poisson regression model yielded several variables positively associated with mean water frogs counts per meter of shore. These were: site (Ratios of means DD/DNP = 2.46 [1.73–3.56], Pr value = 8.61e-07) and within sites waterbody differences (see Supplementary Figure S2), average air temperature of the day of the visit (Ratios of means = 1.03 [1.02–1.05], Pr value = < 2.77e-06), the proportion of animals infected with group I CMTV-NL from total animals caught (Ratios of means = 2.35 [1.25–4.43], Pr value = 0.01), the proportion of animals infected with group III CMTV-NL from total animals caught (Ratios of means = 6.38 [2.67–15.30], Pr value = 3.09e-05), and presence of ranavirus-infected dead animals (Ratios of means = 1.94 [1.51–2.50], Pr value = 1.71e-07). The model with the best fit excluded the variables: presence of fish, water temperature, and presence of ranavirus in the water at a given visit.

Two independent variables of significance were identified in the forward stepwise logistic regression model which had a positive correlation with the ranavirus-positive animals caught. These were the presence of fish (Oda Ratio = 9.15 [2.53–58.70], Pr value = 0) and the average air temperature of the day of the visit (Odds Ratio = 0.75 [0.62 to 0.88], Pr value = 0). The rest of the variables were all discarded by the model. The model details and results are available in Supplementary Figure S2.

## Discussion

The goals of this study were to characterize emerging Dutch ranavirus groups based on their phylogenetic relationship to other fully sequenced CMTV ranaviruses, and to obtain insights in the correlation between distinct genotype features with in vitro growth kinetics and/or effects in wild host populations.

Complete genome sequencing revealed at least three ranavirus groups belonging to the CMTV clade in the Netherlands. Unlike the Dutch ranaviruses belonging to group I, those belonging to group II and III appeared to share a more recent common ancestor with European or Asian strains of amphibian-associated ranaviruses including *T. hermanni* ranavirus (THR), *Rana esculenta* virus (REV) and *Pelophylax esculentus* isolate PEV_DK1, *A. davidianus* ranavirus (ADRV) and Chinese giant salamander iridovirus (CGSIV). All of these viruses and one American ranavirus, *Rana catesbeiana* virus I isolate RCV-Z, grouped together with CMTV-E and pike perch iridovirus (PPIV) within the CMTV clade of ranaviruses. This finding is supportive of the notion that most CMTV-like ranaviruses including all Dutch ranaviruses may represent original European ranavirus strains^3^. CMTV ranaviruses that have been found outside Europe, specifically RCV-Z, ADRV, and CGSIV, have only been reported in captive animal populations^18, 23, 29^ which suggests that international trade has played a role in the emergence of these viruses. This is supported by recent work suggesting that the close phylogenetic relationship between RCV-Z and ADRV could be associated with trade of bullfrogs from the United States to China^29^.

To study potential differences in virulence amongst closely related virus groups complete genome sequencing or partial sequencing of specific virulence-associated genes has proven to be a successful approach. In the United States, a study on ATV strains revealed that those with higher virulence were associated with positive purifying selection for two genes known to be involved in immune-evasion and host immune-suppression (genes eIF-2a and beta-OH-steroid oxidoreductase)^30^. Another study identified a group of truncated genes (49/50L, 65L, 66L, and 87L) that correlated with lower virulence seen in vivo for a suspected less virulent field strain of *Frog virus 3* (FV3 SSME)^28^. The only core iridoviral protein shown to have a shortened ORF in groups II and III was the gene coding for the myristoylated membrane protein 2L. This protein is known to play an important role in enveloping infectious virions and anchoring them into the host cell membrane^25, 31^. This gene, along with genes rich in tandem repeats, have been known to be subject to positive selection in ranaviruses^2, 25, 32^. Moreover, the genes 2L and gene 67L were predicted to result in increased compatibility between the virus and its host after host switching^2^. The later hypothesis was not supported by this study, as genes 2L and 67L of group I isolates from different species were identical, while these genes originating from the same host species sometimes differed. For CMTV NL group III, truncations in two other functional genes were observed; p31K, a delayed early gene involved in transcriptional DNA/RNA synthesis^33^, and a putative integrase-like gene, which permits the incorporation of viral genetic material into host DNA^34^. Whether those, or other not yet annotated, genes account for suspected attenuation or reduced virulence of CMTV-NL group III ranaviruses, warrants further research.

Regarding the investigation on phylo-geographical clustering and short-term evolution of closely related groups, no association could be made between the number of mutations among closely related CMTV-NL group I viruses and the year of detection or distance to index site. Exploration on the repetitive regions of one of the genes studied (17L) revealed that it had the best correlation between number of tandem repeats and common site of origin, with only four variants in the number of repeats among all investigated groups. For gene 62R (neurofilament triplet H1-like gene), the variability was much higher. This may be explained by evidence of recombination reported in this gene^25^. Despite the sequence variability of tandem repeats in gene 67L, the number of repeats for group II and III is low in comparison to group I ranaviruses. Previous work has shown that gene 67L has been subject to positive selection in ranaviruses^2^, which opens up the possibility that distinct profiles in the repeat variation of this gene could serve as additional indicators of the differences in virulence between CMTV-NL groups I, II, and III. More highly variable regions need to be assessed for their utility in discrimination among closely related virus strains in future monitoring studies.

The increasing numbers of captured juveniles during the last visits of the monitoring period at all ponds in DD and at DNP-III is consistent with what is expected for seasonal population dynamics of water frogs from the Netherlands^35^. However at DNP-I and DNP-II, juveniles and metamorphs were only rarely caught, which could indicate a general failure in reproduction at these ponds. Given that these ponds were suitable for water frogs prior to the outbreak in 2010 and that these were the most severely affected by ranavirus during that period, it might be that the decrease of post-metamorphic life stages is associated with CMTV-NL I.

The statistical analysis of the monitoring study revealed that the mean number of water frogs counted per meter shoreline was significantly and positively associated with the proportion of ranavirus-positive swabbed animals and with finding dead ranavirus-positive specimens. This can be explained by detection probability (more likely to detect infected specimens when many frogs are seen and can be caught) and possibly to the density dependence of ranavirus infection^36^. The ratio of the mean counts of frogs for CMTV-NL I was lower than that for CMTV-NL III. This indicates that an increase in proportion of swabbed specimens positive for CMTV-NL I is associated with lower mean counts per meter shoreline than an increase in proportion of swabbed specimens positive for CMTV-NL III. In other words, while ranavirus presence is positively associated with finding water frogs, there is a smaller increase in numbers counted observed with CMTV-NL I than with CMTV-NL III. Consistent with this outcome was the general absence of juveniles and metamorphs observed at DNP-I and DNP-II. Additionally, mortality at DNP, while not massive, did remain constant and involved all life stages of water frogs throughout the visits. Constant CMTV-NL I-associated mortality was also observed in water frogs at DNP during the monitoring period from 2011^37^ and in populations of spadefoot toad larvae and smooth newts at another northern location in a later study^38^.

Our preliminary field data suggest that CMTV-NL group I could be more pathogenic than CMTV-NL III. However, differences in environmental factors were not controlled for and might well contribute to the observed differences in wild frog populations. It has been reported that identical CMTV genotypes can result in differences in disease in amphibians^39^, or that seemingly avirulent ranavirus strains can be quiescent in host amphibian communities and emerge as highly virulent upon reactivation under stressing circumstances^40^. There are only scarce reports on distinct ranavirus strains or groups with suspected differences in virulence for the same host in a country; with many cases comparing strains from nature to others obtained from captive settings^28, 41^. Ultimately, differences in virulence between viruses can only be fully evaluated in closed, controlled, and replicative systems.

In this study, the mean counts of water frogs per meter of shore was influenced by the location of the waterbody (with the DD region favoring higher mean numbers than in DNP) and average air temperature, which is consistent with the ecology of the host. Amphibians tend to metamorphose faster under warm temperatures which could result in overall higher counts of post metamorphic life-stages of water frogs^42^. That conditions seem to be more favorable in certain ponds could also be related to other environmental factors not accounted for in the model, such as nutrient availability. The presence of fish did not appear to influence the mean numbers of water frogs counted per meter of shore but it did have a positive correlation with ranavirus-positive amphibians caught. Previous work has shown that stress-induced by predators could result in immunosuppression and therefore predispose the host to infection by a pathogen^43^. Additionally, fish might be important reservoirs for ranavirus infections in ecosystems as they have been shown to harbor subclinical infections that present a severe risk for amphibian populations^10, 44^. The number of ranavirus-infected amphibians was positively correlated with air temperature of the day, which is known to have a distinctive impact on the outcome of ranavirus infections^41, 45^.

In some of the visits following those in which ranavirus-infected specimens were found, ranavirus was detected in the water but no longer in amphibian hosts. Rapid viral clearance by immunocompetent adult hosts could explain this observation. Successful clearance of ranavirus infections has been described to take place in around 14 days for other amphibian species^46^ which is the amount of time elapsed between the visits. In two of the ponds (DDII and DDIII), ranavirus was found in the water in one occasion (June 15th) but ranavirus-positive animals were never found. It is possible that ranavirus infections in these ponds were restricted to non-sampled, less immune-competent life stages like larvae^47^.

In conclusion, the three Dutch groups of CMTV-NL cluster closely with other European strains of CMTV ranaviruses and all were associated with negative effects on water frog abundance in the wild. However the numbers and mortality patterns of affected host populations appear to differ at sites where distinct CMTV-NL groups occur; in addition, environmental factors other than ranavirus presence are likely to play a role. In vivo challenge trials focused on studying the pathogenesis of these viruses in its natural amphibian host may reveal whether a difference in virulence exists among the distinct CMTV-NL ranavirus groups.

## Materials and methods

### Virus isolation

Tissues from 13 amphibians from ten sites in the Netherlands were selected for virus isolation. Samples originated from native amphibian species submitted dead to the DWHC in the period of 2011–2016. These included: water frogs (*n* = 10), smooth newts (*n* = 2), and a common spadefoot toad (*Pelobates fuscus*; *n* = 1). All selected animals tested positive for ranavirus via conventional PCR using diagnostic primers for the major capsid protein^48^. Information regarding virus isolates, including isolate number, province of origin, GenBank accession numbers, host and coordinates and a map of the collection locations can be found in Supplementary Table S4 and Supplementary Figure S3.

Available organs (kidney, liver, skin, etc.) of each animal were pooled, grinded, and used to make a 10% suspension in Eagle’s minimum essential medium (Gibco) containing 1% antibiotics. The suspension was stored at 4 °C overnight, centrifuged during 10 min at 800 g at 4 °C, filtered (0.45 µm) and inoculated on a confluent layer of epithelioma papullosum cyprinii cells (EPC; ATCC 2872) in T75 flasks. One isolate from 2016 was grown on zebra fish endothelial cells (ZENDO)^49^, supplied with L-15 growth medium (Gibco). When cytopathic effect was observed, the supernatant of the flasks was centrifuged for 10 min at 300 g at 4 °C, and purified via 36% sucrose cushion ultracentrifugation as reported previously^9^.

### Whole genome sequencing and annotation

The DNAeasy Blood and Tissue Kit from Qiagen was used to extract the DNA. The DNA was sheared by sonication and libraries were prepared using KAPA library preparation kit (KAPA Biosystems). A MiSeq running v3 chemistry platform (Illumina) was used to generate 2 × 300 nt paired-end sequence reads. After quality control of the sequence reads using Trim Galore (https://github.com/FelixKrueger/TrimGalore)^50^ de novo assembly using SPAdes produced contigs ranging from 106 to 108 kilobase pairs with an overall average G + C content of 55.28%.

All annotations were performed manually with the aid of the software ORF finder (https://www.ncbi.nlm.nih.gov/orffinder/) to predict the location of coding open reading frames, using the genome of CMTV isolate *P.kl. esculentus*/2013/NL (Genbank accession no. KP056312). All virus genome sequences were submitted to GenBank and are listed in Supplementary Table S1. Overall nucleotide identity was determined using the software Pairwise Genome Comparison, (http://www.ncbi.nlm.nih.gov/sutils/pasc/).

### Phylogenetic analysis

For phylogenetic characterization of the genome sequences of the 13 Dutch ranavirus isolates, complete genome sequences of 25 other members of the family *Iridoviridae* were retrieved from Genbank. The DNA sequences of 50 genes common to all amphibian-associated ranaviruses were aligned using the software MAFFT version 7 with default settings (https://mafft.cbrc.jp/alignment/software/). Maximum likelihood phylogeny was reconstructed with the software MEGA 6.0 using 1000 bootstrap replicates. The best-fit model Generalized + Time Reversible + Gamma distribution + Invariant sites (GTR + G + I) with five discrete gamma categories) was chosen based on the lowest Bayesian Information Criterion score.

### Analysis of genomic mutations and repetitive DNA sequences

The software DNAsp version 5.10 (http://www.ub.edu/dnasp/index_v5.htmlw) was used to perform pairwise comparison of whole genome sequences of CMTV-NL ranaviruses to identify the mutations among all CMTV-NL group I ranaviruses, including common midwife toad ranavirus isolate *P.kl*. *esculentus*/2013/NL^9^. Comparisons were made against the group I isolate which was closest in distance to the index site UU311092007 (7 km).

Out of the 102 annotated genes, the ones with highest variation and rich in repetitive regions were chosen for studying the correlation between the number of tandem repeats and the phylogeographical clustering and virulence. Analysis of the repetitive regions was performed in the 13 fully sequenced ranavirus isolates and in 20 additional sequences of non-fully sequenced ranaviruses originating from four sites (5 specimens per site; 3 sites with group I viruses and 1 site with group III virus). These four sites were chosen on the basis of availability of at least five ranavirus-positive animals per outbreak. Genes rich in repetitive sequences including 17L (hypothetical protein), 62R (neurofilament triplet H1-like protein) and 67L (hypothetical protein) were selected and conventional PCR was performed using primers 17L-FW (5′-GCT CTG GGG TCT TGG GTT TT-3′), 17L-RV (5′-TGG CGG TAA ACA GTC TGA CA-3′), 62R-FW (5′-CGC AAT TCT GGA TGT TCG GT-3′), 62R-RV (5′-GCC GAC TCT ATC CCG TTG TA-3′) 67L-FW (5′-CGT GGC TGG AAG AGA ACT GT-3′), and 67L-RV (5′-GCT GTA CCT GTC TCT CGT GT-3′). The number of tandem repeats was determined via direct Sanger sequencing (Macrogen, the Netherlands).

### In vitro growth kinetics

ZENDO cells were grown at 27 °C and plated at a density of 1 × 10^6^/10 cm^2^ wells. The following day, cells were inoculated with CMTV-NL group I, II, and III at a MOI of 0.01 and supernatant was collected at days 1, 2, 3, 4, 5 and 6 days post infection. Experiments were performed in triplicate. Titrations were performed on 96 well plates by evaluating tissue culture infectious dose 50% (TCID_50_) at 6 days post infection using the Spearman Käarber method^51^.

To analyze whether significant differences existed among the growth patterns among the three viruses in the multi-step growth curve assay, a pairwise comparison of the mean titer values of the three viruses was analyzed using an unpaired *t*-test.

### Field monitoring

A three month long-monitoring study was conducted simultaneously in waterbodies located in the north and south of the country where CMTV-NL groups I and III were known to occur, in order to investigate presence and dynamics of ranavirus infection in amphibian populations. The water frog from which the group II virus was isolated, was found dead on private grounds and neither this site nor its surroundings were included in the monitoring study.

The study area in the north was Dwingelderveld National Park (DNP) located in the province of Drenthe, and the one in the south was a natural area, De Driestruik (DD), located in the province of Limburg. The first recorded ranavirus outbreak in wild amphibians in the Netherlands occurred in a pond in DNP in 2010, caused by a virus belonging to the CMTV-NL group I, and ranavirus-associated mortality has been detected in DNP since then^8, 37^. In DD a ranavirus-associated die-off occurred in 2014, affecting adult water frogs and common spadefoot toad larvae^52^, but there was no evidence for ranavirus-associated mortality in 2015. The waterbodies monitored in 2016 consisted of three ponds in DD and two ponds and one fen in DNP. Ranavirus was previously shown to occur at all waterbodies from DNP^37^, whereas at DD, it was only known to be present in two waterbodies (DD-II and DD-III)^52^. However, a third pond at DD (DD-I) was in close proximity (300–900 m) to the affected ponds and made it a likely target for ranavirus spread mediated by amphibians, birds, or humans. Information regarding coordinates of waterbodies and distance among sampled sites can be found in Supplementary Figure S4. Both study areas were open to the public during the surveillance period in 2016.

The monitoring took place during the summer months (June–August) when different life-stages of water frogs can be found in the water. During this period there can be increased contact among conspecifics or cannibalism, known to be favorable factors for ranavirus infection and spread^53^. In an earlier study, the water frog was found to be the species most affected by ranavirus in the Netherlands^22^. Animals of this species have been found dead and are known to be susceptible to infection by any of the three groups of CMTV-NL. Both DNP and DD were monitored simultaneously every two weeks in 2016, i.e., on seven occasions. Population counts were performed using a previously established protocol^37^. For counts along the shoreline, ponds were fully circled and in the case of the fen only partly, due to lack of full access. To estimate the number of (sub-)adult water frogs per shore meter, total number of adult water frogs counted was divided by the perimeter of the pond. In the case of the fen DNP-III, the number of water frogs observed at a given time was divided by the distance in meters from the monitored transect.

Water frogs (*n* = 327), smooth newts (*n* = 68), common frogs (*n* = 30), and common toad (*Bufo bufo*; *n* = 2) were counted, and the post metamorphic life stages collected and sampled by skin-swabbing at both study areas. The three water frog species (*Pelophylax lessonae, Pelophylax ridibundus,* and the hybrid “species” *P.kl*. *esculentus*) share a genetic background^54^ can be difficult to identify on species level based on morphology, and were therefore pooled together as *Pelophylax* spp. for data analysis. Other amphibian species that were sampled included the crested newt (*Triturus cristatus; n* = 8), in DNP and the alpine newt (*n* = 3) in DD. Swabbing of larval stages was generally avoided to prevent the development of stress-induced infections^47^. The larvae of crested newts were also sampled; since their large body size (around 4 cm prior to metamorphosis)^55^ made them more likely to withstand the stress of handling. Collected specimens were kept in individual plastic bags filled with water, and once all animals had been swabbed, these were released back to the pond they were captured from (exemption permit no. FF/75 A/2016/015). Cross contamination was prevented by changing vinyl powder-free gloves after each capture. The skin of the animals was swabbed once all over the body with a sterile, cotton-tipped swab (Copan), and the species and life stage were recorded. Numbers counted and species of all swabbed amphibians can be found in Supplementary Table S2A for DNP and Supplementary Table S2B for DD.

Any dead amphibians found at the sites were preserved in plastic bags surrounded by cooling gel packs. Tissues from all dead specimens were stored at −80 °C. In the case of sufficiently fresh specimens, some of the tissues (liver, kidney, skin, spleen) were fixed in 10% formalin for histopathological assessment. The collection of dead frogs was done under permit no. FF/75 A/2008/075; as it is not considered an animal experiment, permission of the Committee on the Ethics of Animal Experiment was not required.

A water sample of 500 mL was taken from each pond during each visit in sterile plastic bottles. The pH and temperature of the water were measured at a distance of ~1 m from the shore of each waterbody, using PANHEPA pH strips and a digital thermometer (Prima Long) as extreme fluctuations in these environmental parameters have been shown to influence the outcome of ranavirus infection^26^. All gear and equipment was disinfected using bleach (1%) or VirkonS^®^ (1%) according to protocol^56^ before sampling the next pond.

### Histopathological and immunohistochemical analysis

Sufficiently fresh specimens that were retrieved from the field in cool packs were afterwards fixed in 10% formalin for at least 24 h and then processed for routine histopathological analysis using hematoxylin/eosin staining and immunohistochemistry. The immunohistochemistry method used is reported elsewhere^22^.

### DNA extraction

DNA was extracted from swabs using the Blood and Tissue Kit (Qiagen). Briefly, cotton tips were incubated for 1 h at 56 °C in 400 µl Lysis buffer with 60 µl of proteinase K (20 mg/ml). After a 10-minute centrifugation step at 7000 g, the resulting solution was processed according to the manufacturer’s protocol. For DNA extraction from water, samples were first defrosted and filtered through Millipore Stericup filters (0.22 µm). The filters were cut into small fragments and DNA was extracted with the DNEasy Power Kit (Qiagen) according to the manufacturer’s protocol.

### Ranavirus quantitative PCR

Ranavirus DNA was detected using SYBR-green quantitative PCR using diagnostic primers for a fragment of the polymerase gene^57^. Each sample was tested in triplicate and negative controls consisting of a distilled water sample and a ranavirus-negative amphibian were included in every run. Standard curves were generated by making 10-fold dilutions from sucrose purified CMTV-NL ranavirus with a known titer. Samples were deemed positive when an amplification curve generated detectable fluorescence above the threshold cycle in two of the three replicates. Samples that tested positive in only one replicate were tested twice to confirm positivity. To rule out false negative results in water samples due to DNase activity, PCR was performed using primers targeting chloroplast mitochondria, known to be ubiquitously present in fresh water ecosystems^58^.

### Ranavirus and *B. dendrobatidis* conventional PCR

To characterize phylogroups, single positive samples from DNP and DD were processed by conventional PCR and Sanger-sequenced with the aid of primers for seven ranavirus genes as described previously^7, 22^. For *Bd* detection, the primers and conditions used were previously described^59^.

### Statistical analysis of the monitoring data

In order to investigate whether the number of water frogs counted per meter of shore was affected by the group of ranavirus occurring at the site or by other external factors, a backward stepwise Poisson regression model was performed and Akaike’s Information Criterion used to select the best-fitting model. Number of water frogs per meter of shore during each visit was considered as a dependent variable, independent variables were: (a) site, (b) waterbody nested within site, (c) proportion of water frogs infected with Group I virus from total numbers caught, d) proportion of water frogs infected with Group III virus from total numbers caught, e) presence of fish, f) presence of dead ranavirus-infected animals, g) water temperature on the visit day, h) average air temperature of the day of visit and i) presence of ranavirus in water at a given visit.

In order to investigate factors that could potentially have an effect on proportion of ranavirus-infected animals from total numbers caught, forward stepwise logistic regression model was performed using Akaike’s Information Criterion. The proportion of ranavirus-infected animals from total numbers caught was the dependent variable. The independent variables were: (a) site (b) waterbody nested within site, (c) presence of fish, (d) proportion of caudates from total numbers of amphibians caught at a given time, (e) the number of amphibian species caught at a given time, (f) the water temperature recorded on the day of the visit, and (g) the average air temperature of the day of visit.

For those effects in the final models, 95% profile log-likelihood confidence intervals for the odds ratios were calculated.

## Electronic supplementary material

Figure S1(TIF 120 kb)

Figure S2(PDF 146 kb)

Figure S3(TIF 217 kb)

Figure S4(TIF 727 kb)

Table S1(DOCX 15 kb)

Table S2a(DOCX 16 kb)

Table S2b(DOCX 24 kb)

Table S3(DOCX 20 kb)

Table S4(DOCX 13 kb)

Supplementary figure legends(DOCX 14 kb)
