# Supplementary figures and images for: Ranavirus genotypes in the Netherlands and their potential association with virulence in water frogs (Pelophylax spp.)
Source: Emerg Microbes Infect. 2018 Apr 4;7:56. doi: 10.1038/s41426-018-0058-5 (PMC5882854; doi:10.1038/s41426-018-0058-5)

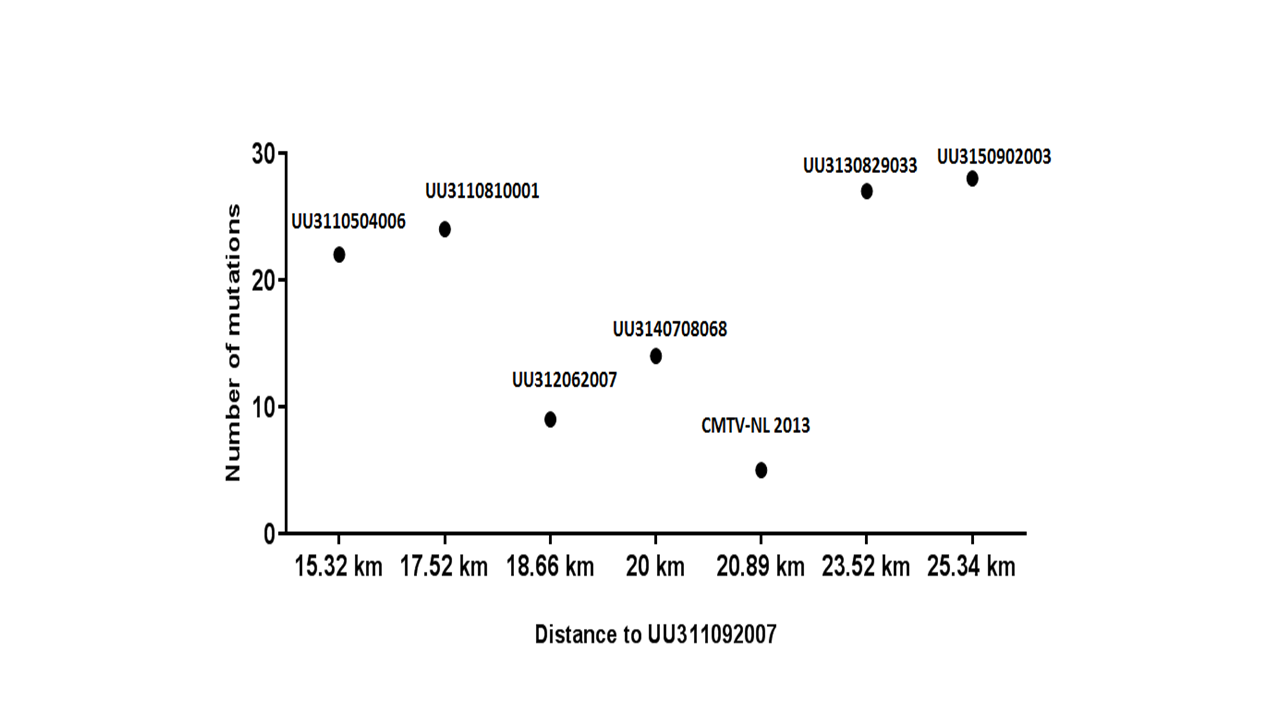

Supplement: Supplementary file 1 — Figure S1(TIF 120 kb) [file 41426_2018_58_MOESM1_ESM.tif]

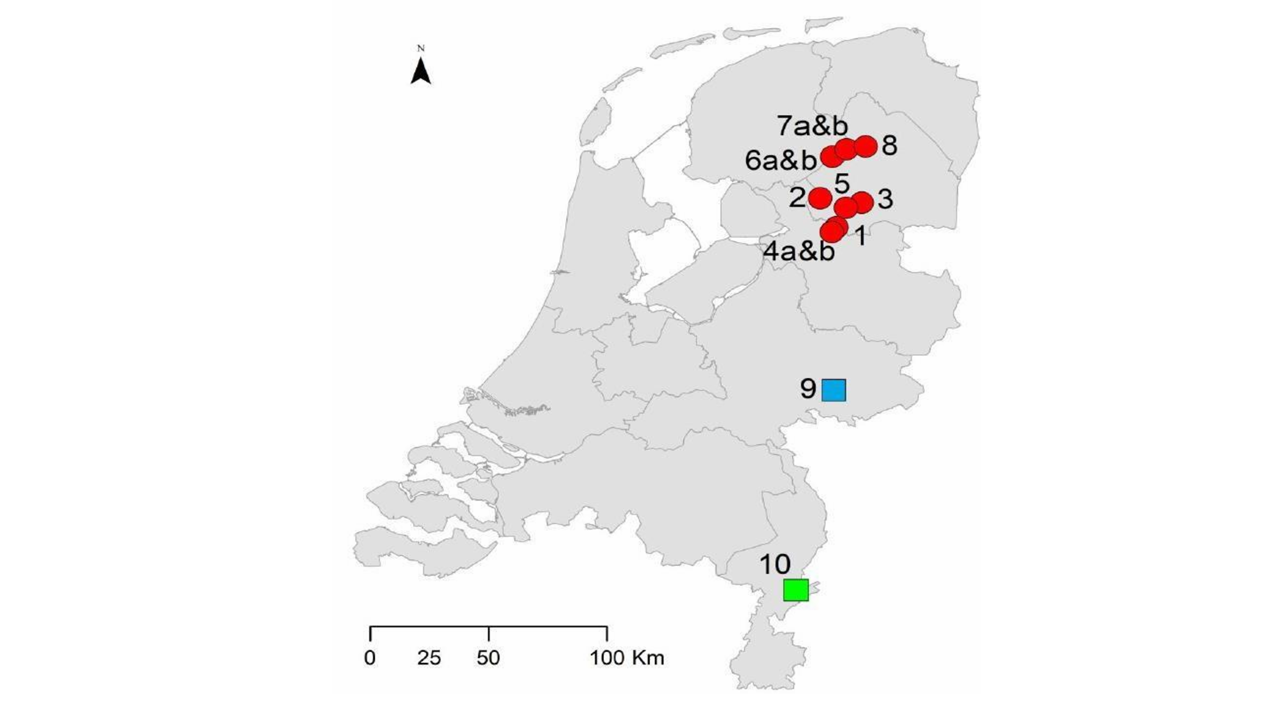

Supplement: Supplementary file 3 — Figure S3(TIF 217 kb) [file 41426_2018_58_MOESM3_ESM.tif]

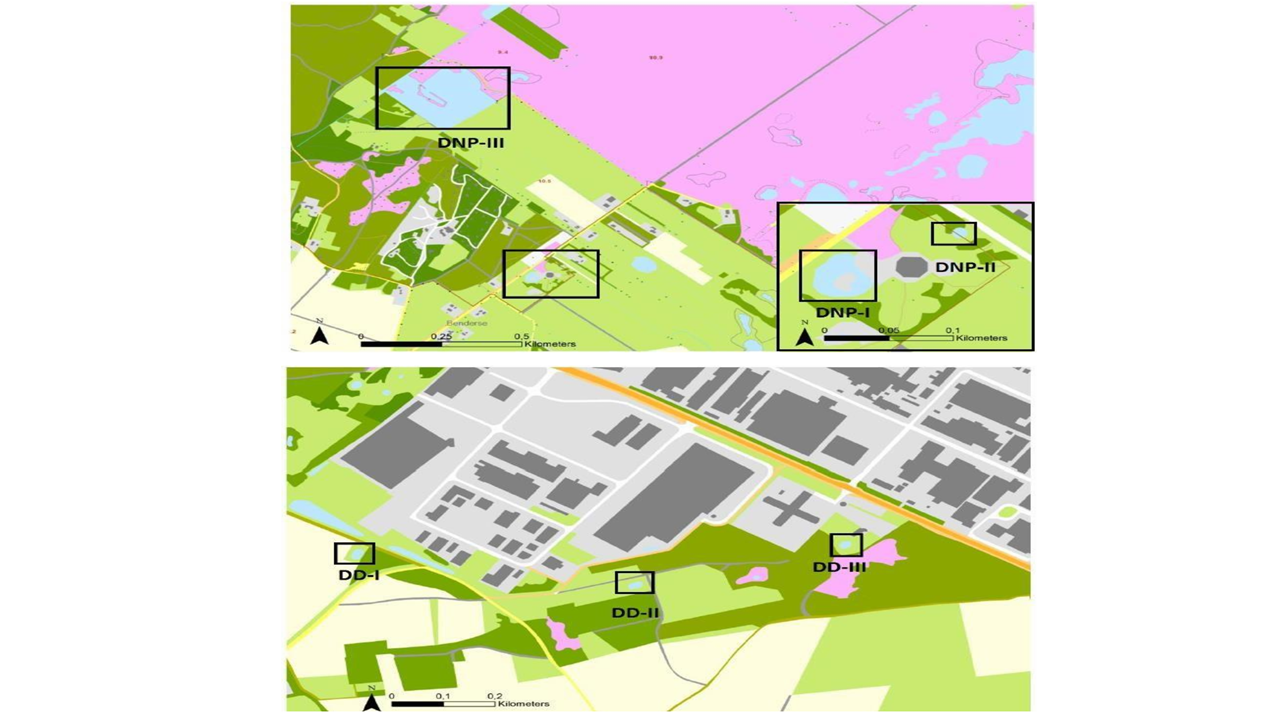

Supplement: Supplementary file 4 — Figure S4(TIF 727 kb) [file 41426_2018_58_MOESM4_ESM.tif]
