# Supplementary material for: Ranavirus genotypes in the Netherlands and their potential association with virulence in water frogs (Pelophylax spp.)
Source: Emerg Microbes Infect. 2018 Apr 4;7:56. doi: 10.1038/s41426-018-0058-5 (PMC5882854; doi:10.1038/s41426-018-0058-5)
Supplement: Supplementary file 2 — Figure S2(PDF 146 kb) [file 41426_2018_58_MOESM2_ESM.pdf]

Supplementary figure S4. Statistical model details

Model 1

========================

A backward stepwise poisson regression was used to analyse the number of pelophylax with the log of meter shore as an offset. Akaike's Information Criterion was used for model reduction.
The independent variables were

- Site
- Waterbody nested within site Ranavirus.water
- Proportion.typeI.virus.
- Proportion.typeIII.virus
- Presence.fish
- Presence.ranavirus.infected.dead
- Water.temp
- Average.air.temp

From those effects in the final model 95% profile log-likelihood confidence intervals for the ratio's of mean counts were calculated.
The final model contained the following independent variable:

Site

Waterbody nested within Site Proportion.typeI.virus.

Proportion.typeIII.virus

Presence.ranavirus.infected.dead

Average.air.temp

Call:

glm(formula = Pelophylax.count ~ factor(Site) + factor(Site):factor(Waterbody) + Proportion.typeI.virus. + Proportion.typeIII.virus + Average.air.temp + factor(Presence.ranavirus.infected.dead), family = poisson)

Deviance Residuals:

Min	1Q	Median	3Q	Max
-7.5750	-4.0956	-0.5925	2.0708	11.6389

Coefficients:

	Estimate	Std. Error	z value	Pr(>|z|)
(Intercept)	1.545822	0.201340	7.678	1.62e-14
factor(Site)De Driestruik	0.902874	0.183472	4.921	8.61e-07
Proportion.typeI.virus.	0.857689	0.322030	2.663	0.00774
Proportion.typeIII.virus	1.853501	0.444817	4.167	3.09e-05
Average.air.temp	0.035574	0.007589	4.687	2.77e-06
factor(Presence.ranavirus.infected.dead)1	0.664903	0.127169	5.229	1.71e-07
factor(Province)DNP factor(site)2	-0.465363	0.245546	-1.895	0.05806
factor(Province)DD:factor(site)2	1.398211	0.110385	12.667	< 2e-16
factor(Province)DNP:factor(site)3	1.556579	0.191487	8.129	4.33e-16
factor(Province)DD:factor(site)3	1.012437	0.114352	8.854	< 2e-16

Signif. codes: 0 *** 0.001 ** 0.01* 0.05 0.1 1

(Dispersion parameter for poisson family taken to be 1)

Null deviance: 1541.49 on 32 degrees of freedom

Residual deviance: 660.01 on 23 degrees of freedom

AIC: 843.48
Number of Fisher Scoring iterations: 5
The estimates in the summary above are log ratio's of the means per meter shore. So in the line factor(Site) De Driestruik the estimate is .90 which is the log of the ratio of the mean count per meter shore of the site De Driestuik devided by the mean count per meter shore of the site Dwingelderveld.
Below the ratio's of the mean counts per meter shore and the confidence intervals are given. So the mean count per meter shore of De Driestruik and Dwingelderveld is 2.47 with a confidence interval of (1.73 3.57)
95% profile log-likelihood confidence intervals for the oddsratio's

	2.5 %	97.5 %	Ratio's
(Intercept)	3.1321117	6.904119	4.691826
factor(Site)De Driestruik	1.7345343	3.567653	2.466682
Proportion.typeI.virus.	1.2533813	4.431925	2.357705
Proportion.typeIII.virus	2.6728199	15.302847	6.382122
Average.air.temp	1.0208484	1.051680	1.036214
factor(Presence.ranavirus.infected.dead)1	1.5190015	2.501786	1.944302
factor(Province)DNP:factor(site)2	0.3837036	1.009431	0.627907
factor(Province)DD:factor(site)2	3.2761561	5.052120	4.047951
factor(Province)DNP:factor(site)3	3.2806781	6.962032	4.742568
factor(Province)DD:factor(site)3	2.2090418	3.460034	2.752300

Model 2
=========
A forward stepwise logistic regression model was used to analyse the number of ranavirus casus of the total number of caught *Pelophylax* using Akaike's Information Criterion. The independent variables were:

- Site
- Waterbody nested within site
- Presence.fish
- Proportion.caudates
- Number.species
- Water.temp
- Average.air.temp

From those effects in the final model 95% profile log-likelihood confidence intervals for the oddsratio's were calculated.

The independent variables in the final model were:

- Presence.fish
- Average.air.temp

95% profile log-likelihood confidence intervals for the oddsratio's:

	2.5 %	97.5 %	OR
factor(Presence.fish)1	2.92757930	8.122494e+01	11.5441210
Average.air.temp	0.60270648	8.926982e-01	0.7502832

Analysis final formula: glm(formula = cbind(Ranavirus, N.Pelophylax - Ranavirus) ~ factor(Presence.fish) + Average.air.temp, family = binomial)

Deviance Residuals:
Min 1Q Median 3Q Max
-3.1336 -0.5855 -0.2685 0.0000 2.4816

Coefficients:
Estimate Std. Error z value Pr(>|z|) (Intercept)
0.23392 1.51419 0.154 0.87722 factor(Presence.fish)1 2.21412
0.76095 2.910 0.00362 ** Average.air.temp -0.28306
0.08866 -3.193 0.00141 **

Signif. codes: 0 *** 0.001 ** 0.01* 0.05 0.1 1

(Dispersion parameter for binomial family taken to be 1)
Null deviance: 76.699 on 33 degrees of freedom

Residual deviance: 43.158 on 31 degrees of freedom
AIC: 66.468
Number of Fisher Scoring iterations: 6

95% profile log-likelihood confidence intervals for the oddsratio's:
2.5 % 97.5 % OR (Intercept)
0.0646676 27.6561277 factor(Presence.fish)1 2.5348983
58.7041444 9.1533654 Average.air.temp 0.6200547
0.8817204 0.7534736
