# Supplementary material for: Ranavirus genotypes in the Netherlands and their potential association with virulence in water frogs (Pelophylax spp.)
Source: Emerg Microbes Infect. 2018 Apr 4;7:56. doi: 10.1038/s41426-018-0058-5 (PMC5882854; doi:10.1038/s41426-018-0058-5)
Supplement: Supplementary file 5 — Table S1(DOCX 15 kb) [file 41426_2018_58_MOESM5_ESM.docx]

**Supplementary Table S1** Number of mutations undergone in CMTV NL I ranaviruses in a period of 5 years

| **Isolate** | 3110504006 | 3110810001 | 3110920007 | 3120627007 | KP056312 | 3130829033 | 3140625035 | 3140708068 | 3150902003 | 3150625026 |
| --- | --- | --- | --- | --- | --- | --- | --- | --- | --- | --- |
| 3110504006 | NA | 12 | 22 | 23 | 16 | 21 | 14 | 14 | 13 | 28 |
| 3110810001 | 12 | NA | 24 | 14 | 14 | 15 | 11 | 11 | 7 | 20 |
| 3110920007 | 22 | 24 | NA | 9 | 14 | 5 | 27 | 27 | 28 | 14 |
| 3120627007 | 23 | 14 | 9 | NA | 31 | 8 | 21 | 22 | 22 | 8 |
| KP056312 | 21 | 15 | 5 | 8 | 22 | NA | 19 | 19 | 14 | 13 |
| 3130829033 | 14 | 11 | 27 | 21 | 24 | 19 | NA | 5 | 8 | 25 |
| 3140625035 | 14 | 11 | 27 | 22 | 22 | 19 | 5 | NA | 5 | 26 |
| 3140708068 | 16 | 14 | 14 | 31 | NA | 22 | 24 | 22 | 13 | 31 |
| 3150902003 | 13 | 7 | 28 | 15 | 16 | 14 | 8 | 5 | NA | 27 |
| 3150625026 | 28 | 20 | 14 | 8 | 31 | 13 | 25 | 26 | 27 | NA |
