# Supplementary material for: Ranavirus genotypes in the Netherlands and their potential association with virulence in water frogs (Pelophylax spp.)
Source: Emerg Microbes Infect. 2018 Apr 4;7:56. doi: 10.1038/s41426-018-0058-5 (PMC5882854; doi:10.1038/s41426-018-0058-5)
Supplement: Supplementary file 6 — Table S2a(DOCX 16 kb) [file 41426_2018_58_MOESM6_ESM.docx]

**Supplementary Table S2A** Monitoring data from Dwingelderveld

| Date of visit |  | Jun 1 | Jun 15 | Jun 29 | Jul 13 | Jul 28 | Aug 10 | Aug 24 |
| --- | --- | --- | --- | --- | --- | --- | --- | --- |
| Air temperature (range/day) (°C) |  | 15.3-24.9 | 11.7-19.0 | 13.2-20.1 | 8.9-18.0 | 12.8-22.2 | 7.3-16.7 | 16.5-30.2 |
| DNP-I site (104 m. shoreline) | Water temperature (°C) | NM | NM | 18.9 | 19.9 | 21.3 | 16.9 | 19.8 |
|  | No. (sub-)adult *Pelophylax* spp. counted | 51 | 8 | 2 | 6 | 10 | 4 | 21 |
|  | No. (sub-)adult *Pelophylax* spp. counted per m. shoreline | 0.49 | 0.08 | 0.02 | 0.06 | 0.10 | 0.04 | 0.20 |
|  | Ranavirus PCR-test results for water | positive | negative | positive | negative | negative | positive | negative |
|  | No. Ranavirus PCR-positive*/*total *Pelophylax* spp. caught (%) | 1/7 (14%) | NA | NA | NA | NA | 0/3 (0%) | NA |
|  | No. Ranavirus PCR-positive */* total no. of *Rana temporaria* caught | 0/3 (0%) | NA | NA | NA | 0/7 (0%) | 0/6 (0%) | 0/7 (0%) |
|  | No. Ranavirus PCR-positive */* total no. of *Bufo bufo* caught | 0/1 (0%) | 1 negative dead | NA | NA | NA | NA | NA |
| DNP-II site (34 m. shoreline) | Water temperature (°C) | ND | ND | 16.8 | 19.9 | 18.8 | 14.2 | 17.3 |
|  | No. (sub-)adult *Pelophylax* spp. counted | 17 | 11 | 11 | 6 | 5 | 4 | 1 |
|  | No. (sub-)adult *Pelophylax* spp. counted per m. shoreline | 0.50 | 0.32 | 0.32 | 0.18 | 0.15 | 0.12 | 0.03 |
|  | Ranavirus PCR-test results for water | negative | positive | negative | negative | positive | positive | negative |
|  | No. Ranavirus PCR-positive */* total no. of *Pelophylax* spp. caught (%) | 1/9 (11%) | 1/4 (25%) | 0/3 (0%) | 0/3 (0%) | NA | NA | NA |
|  | No. Ranavirus PCR-positive*/* total no. of *Triturus cristatus* caught (%) | NA | NA | 0/3 (0%) | NA | 1/8 (12.5%) | 0/6 (0%) | 0/2 (0%) |
|  | No. Ranavirus PCR-positive */* total no. of *Rana temporaria* caught (%) | 0/2 (0%) | NA | NA | NA | NA | 0/2 (0%) | 0/1 (0%) |
|  | No. Ranavirus PCR-positive*/* total no. of *Lissotriton vulgaris* caught (%) | 0/8 (0%) | 0/5 (0%) | NA | NA | NA | NA | NA |
| DNP-III site (150 m. shoreline) | Water temperature (°C) | ND | ND | 19.8 | 21.0 | 21.1 | 21.7 | 22.3 |
|  | No. (sub-)adult *Pelophylax* spp. counted | 162 | 44 | 72 | 52 | 91 | 95 | 34 |
|  | No. (sub-)adult *Pelophylax* spp. counted per m. shoreline | 1.08 | 0.29 | 0.48 | 0.35 | 0.61 | 0.63 | 0.23 |
|  | Ranavirus PCR-test results for water | positive | negative | positive | positive | negative | positive | negative |
|  | No. Ranavirus PCR-positive */* total no. of *Pelophylax* spp. caught (%) | 1/7 (14%) | 0/8 (0%) | 1/3 (33%) and 3 positive dead | 6/19 (32%) and 3 positive dead | 0/14 (0%) | 0/15 (0%) and 1 positive dead | 0/7 (0%) |
|  | No. Ranavirus PCR-positive */* total no. of *Lissotriton vulgaris* caught (%) | NA | NA | NA | 1/1(100%) | NA | 0/1 (0%) | NA |
| NA. Not applicable indicates this species was not caught that day. NM indicates that the water temperature was not measured that day. | | | | | | |  |  |
