# Supplementary material for: Ranavirus genotypes in the Netherlands and their potential association with virulence in water frogs (Pelophylax spp.)
Source: Emerg Microbes Infect. 2018 Apr 4;7:56. doi: 10.1038/s41426-018-0058-5 (PMC5882854; doi:10.1038/s41426-018-0058-5)
Supplement: Supplementary file 7 — Table S2b(DOCX 24 kb) [file 41426_2018_58_MOESM7_ESM.docx]

**Supplementary Table S2B** Monitoring data from De Driestruik

| Date of visit |  | Jun 1 | Jun 15 | Jun 29 | Jul 13 | Jul 28 | Aug 10 | Aug 21 |
| --- | --- | --- | --- | --- | --- | --- | --- | --- |
| Air temperature (range/day) (°C) |  | 13.2-23.1 | 12.1- 20.0 | 13.5-21.4 | 10.5-20.5 | 16.5-24.6 | 7.1-18.7 | 16.4-32.2 |
| DD-I site (47 m. shoreline) | Water temperature (°C) | 17.5 | 21.0 | 21.6 | 19.8 | NM | 20.3 | 26.6 |
|  | No. (sub-)adult *Pelophylax* spp. counted | 4 | 12 | 12 | 6 | 41 | 84 | 77 |
|  | No. (sub-)adult *Pelophylax* spp. counted per m. shoreline | 0.09 | 0.26 | 0.26 | 0.13 | 0.87 | 1.79 | 1.64 |
|  | Ranavirus PCR-test results for water | negative | negative | negative | positive | negative | positive | positive |
|  | No. Ranavirus PCR-positive*/*total *Pelophylax* spp. caught (%) | 0/4 (0%) | 0/6 (0%) | 0/6 (0%) and 1 negative dead | 1/4 (25%) | 0/18 (0%) | 9/18 (50%) and 1 positive dead | 0/15 (0%) |
|  | No. Ranavirus PCR-positive */* total no. of *Lissotriton vulgaris* caught | 0/2 (0%) | NA | 0/1 (0%) and 1 negative dead | NA | NA | NA | NA |
| DD-II site (94 m. shoreline) | Water temperature (°C) | 17.0 | 21.0 | 25.6 | 21.5 | NM | 20.9 | 27.0 |
|  | No. (sub-)adult *Pelophylax* spp. counted | 32 | 81 | 36 | 32 | 167 | 195 | 152 |
|  | No. (sub-)adult *Pelophylax* spp. counted per m. shoreline | 1 | 2.53 | 1.13 | 1 | 5.22 | 6.09 | 4.75 |
|  | Ranavirus PCR-test results for water | negative | positive | negative | negative | negative | negative | negative |
|  | No. Ranavirus PCR-positive */* total no. of *Pelophylax* spp. caught (%) | 0/10 (0%) | 0/15 (0%) | 0/7 (0%) | 0/2 (0%) and 1 negative dead | 0/16 (0%) | 0/19 (0%) | 0/17 (0%) |
|  | No. Ranavirus PCR-positive*/* total no. of *Ichtyosaura alpestris* caught (%) | 0/1 (0%) | NA | NA | NA | NA | NA | NA |
|  | No. Ranavirus PCR-positive */* total no. of *Rana temporaria* caught (%) | 0/2 (0%) | NA | NA | NA | NA | NA | NA |
|  | No. Ranavirus PCR-positive*/* total no. of *Lissotriton vulgaris* caught (%) | 0/10 (0%) | 0/12 (0%) | 0/4(0%) | 1 negative dead | NA | NA | NA |
|  | No. Ranavirus PCR-positive*/* total no. of *Bufo bufo* caught (%) | NA | 1 negative dead | NA | NA | NA | NA | NA |
| DD-III site (35 m. Water temperature (°C) shoreline) |  | 17.2 | 21.0 | 24.8 | 22.6 | NM | 20.1 | 29.3 |
|  | No. (sub-)adult *Pelophylax* spp. counted | 35 | 16 | 37 | 27 | 87 | 123 | 121 |
|  | No. (sub-)adult *Pelophylax* spp. counted per m. shoreline | 1 | 0.46 | 1.06 | 0.77 | 2.49 | 3.51 | 3.46 |
|  | Ranavirus PCR-test results for water negative positive | negative | positive | negative | negative | negative | negative | negative |
|  | No. Ranavirus PCR-positive */* total no. of *Pelophylax* spp. caught (%) | 0/15 (0%) | 0/5 (0%) | 0/8 (0%) | 0/1 (0%) | 0/11 (0%) | 0/12 (0%) and 1 positive dead | 0/16 (0%) |
|  | No. Ranavirus PCR-positive */* total no. of *Lissotriton vulgaris* caught (%) | 0/10 (0%) | 0/12 (0%) | 0/2 (0%) | NA | NA | NA | NA |
|  | No. Ranavirus PCR-positive*/* total no. of *Ichtyosaura alpestris* caught (%) | 0/2 (0%) | NA | NA | NA | NA | NA | NA |
|  | No. Ranavirus PCR-positive*/* total no. of *Bufo bufo* caught (%) | NA | NA | NA | 0/1 (0%) | NA | NA | NA |
| NA. Not applicable indicates this species was not caught that day. NM indicates that the water temperature was not measured that day. |  | | | | | | |  |
