# Supplementary material for: Ranavirus genotypes in the Netherlands and their potential association with virulence in water frogs (Pelophylax spp.)
Source: Emerg Microbes Infect. 2018 Apr 4;7:56. doi: 10.1038/s41426-018-0058-5 (PMC5882854; doi:10.1038/s41426-018-0058-5)
Supplement: Supplementary file 8 — Table S3(DOCX 20 kb) [file 41426_2018_58_MOESM8_ESM.docx]

**Supplementary Table S3** Life stages and lengths of captured water frogs

|  | visit 1 | visit 2 | visit 3 | visit 4 | visit 5 | visit 6 | visit 7 |  |  | visit 1 | visit 2 | visit 3 | visit 4 | visit 5 | visit 6 | visit 7 |
| --- | --- | --- | --- | --- | --- | --- | --- | --- | --- | --- | --- | --- | --- | --- | --- | --- |
| DD-I | 7 cm | 5.5 cm | 4.5 cm | 5.5 cm | 3 cm | 2.6 cm | 2.1 cm |  | DNP-I | 3.2 cm |  |  |  |  |  | 2.4 cm |
|  | 9 cm | 7 cm | 5.5 cm | 5.4 cm | 3.2 cm | 5.6 cm | 2.5 cm |  |  | 3.5 cm |  |  |  |  |  | 3.2 cm |
|  | 5 cm | 6 cm | 7.5 cm | 8 cm | 2.5 cm | 4 cm | 2.2 cm |  |  | 3.6 cm |  |  |  |  |  | 3.3 cm |
|  | 3.5 cm | 3.5 cm | 4 cm | 4.1 cm | 3.1 cm | 2.4 cm | 4 cm |  |  | 5 cm |  |  |  |  |  |  |
|  |  | 8 cm | 6 cm |  | 3.2 cm | 3.5 cm | 3.1 cm |  |  | 3 cm |  |  |  |  |  |  |
|  |  | 5.5cm | 6 cm |  | 2.1 cm | 2.3 cm | 2.4 cm |  |  | 4.5 cm |  |  |  |  |  |  |
|  |  |  | 9 cm |  | 2.9 cm | 2.4 cm | 4.2 cm |  |  | 3.5 cm |  |  |  |  |  |  |
|  |  |  |  |  | 2.5 cm | 2.3 cm | 2.7 cm |  |  |  |  |  |  |  |  |  |
|  |  |  |  |  | 2.6 cm | 4 cm | 2.3 cm |  |  |  |  |  |  |  |  |  |
|  |  |  |  |  | 3.2 cm | 2.7 cm | 2.2 cm |  |  |  |  |  |  |  |  |  |
|  |  |  |  |  | 2.1 cm | 2.3 cm | 2.6 cm |  |  |  |  |  |  |  |  |  |
|  |  |  |  |  | 2.5 cm | 2.6 cm | 2.5cm |  |  |  |  |  |  |  |  |  |
|  |  |  |  |  | 2.2 cm | 2.7 cm | 2.3 cm |  |  |  |  |  |  |  |  |  |
|  |  |  |  |  | 2.2 cm | 3 cm | 2.1 cm |  |  |  |  |  |  |  |  |  |
|  |  |  |  |  | 2.5 cm | 2.5 cm | 2.5cm |  |  |  |  |  |  |  |  |  |
|  |  |  |  |  | 2.7 cm | 8 cm |  |  |  |  |  |  |  |  |  |  |
|  |  |  |  |  | 5.2 cm | 8.1 cm |  |  |  |  |  |  |  |  |  |  |
|  |  |  |  |  | 5.8 cm | 8.2 cm |  |  |  |  |  |  |  |  |  |  |
|  |  |  |  |  |  |  |  |  |  |  |  |  |  |  |  |  |
|  | visit 1 | visit 2 | visit 3 | visit 4 | visit 5 | visit 6 | visit 7 |  |  | visit 1 | visit 2 | visit 3 | visit 4 | visit 5 | visit 6 | visit 7 |
| DD-II | 3 cm | 5 cm | 6 cm | 2.7 cm | 3 cm | 6.1 cm | 3.1 cm |  | DNP-II | 3.5 cm | 4.5 cm | 6.5 cm | 3 cm |  |  |  |
|  | 5.5 cm | 3.5 cm | 5 cm | 8.5 cm | 3 cm | 6 cm | 3.5 cm |  |  | 5 cm | 4 cm | 4.2 cm | 2.5 cm |  |  |  |
|  | 5 cm | 5.5 cm | 4.5 cm |  | 4.2 cm | 3 cm | 3.2 cm |  |  | 3.5 cm | 4 cm | 3.7 cm | 2.5 cm |  |  |  |
|  | 5.5 cm | 6.5 cm | 4.5 cm |  | 5.6 cm | 3 cm | 5.3 cm |  |  | 3 cm | 4 cm |  |  |  |  |  |
|  | 3 cm | 5.3 cm | 4 cm |  | 5.5 cm | 3.2 cm | 3.5 cm |  |  | 4 cm |  |  |  |  |  |  |
|  | 4 cm | 5 cm | 3.5 cm |  | 3.1 cm | 6 cm | 3.3 cm |  |  | 3.5 cm |  |  |  |  |  |  |
|  | 5 cm | 4 cm | 6 cm |  | 3 cm | 3 cm | 3.2 cm |  |  | 3 cm |  |  |  |  |  |  |
|  | 5 cm | 6 cm |  |  | 3 cm | 2.5 cm | 2.4 cm |  |  | 3.5 cm |  |  |  |  |  |  |
|  | 2.5 cm | 3.5 cm |  |  | 3.2 cm | 3.1 cm | 3 cm |  |  | 3.2 cm |  |  |  |  |  |  |
|  | 4 cm | 4.5 cm |  |  | 3 cm | 3.5 cm | 3 cm |  |  |  |  |  |  |  |  |  |
|  |  | 4.5 cm |  |  | 2.9 cm | 3 cm | 2.9 cm |  |  |  |  |  |  |  |  |  |
|  |  | 3.9 cm |  |  | 2.9 cm | 2.2 cm | 5.5 cm |  |  |  |  |  |  |  |  |  |
|  |  | 3.2 cm |  |  | 2.4 cm | 5.5 cm | 3.1 cm |  |  |  |  |  |  |  |  |  |
|  |  | 2.8 cm |  |  | 3 cm | 3 cm | 3.2 cm |  |  |  |  |  |  |  |  |  |
|  |  | 4.5 cm |  |  | 3.1 cm | 2.5 cm | 2.8 cm |  |  |  |  |  |  |  |  |  |
|  |  |  |  |  | 2.7 cm | 3.1 cm | 3.1 cm |  |  |  |  |  |  |  |  |  |
|  |  |  |  |  |  | 3 cm | 3.2 cm |  |  |  |  |  |  |  |  |  |
|  |  |  |  |  |  | 2.6 cm |  |  |  |  |  |  |  |  |  |  |
|  |  |  |  |  |  | 2.6 cm |  |  |  |  |  |  |  |  |  |  |
|  |  |  |  |  |  |  |  |  |  |  |  |  |  |  |  |  |
|  | visit 1 | visit 2 | visit 3 | visit 4 | visit 5 | visit 6 | visit 7 |  |  | visit 1 | visit 2 | visit 3 | visit 4 | visit 5 | visit 6 | visit 7 |
| DD-III | 4 cm | 5 cm | 4.3 cm | 4.3 cm | 2.8 cm | 2.6 cm | 2.9 cm |  | DNP-III | 4 cm | 4 cm | 4.1 cm | 2.5 cm | 2.7 cm | 2.7 cm | 2.5 cm |
|  | 3.2 cm | 4 cm | 5.5 cm |  | 3 cm | 2.9 cm | 2.3 cm |  |  | 5.3 cm | 3.3 cm | 3.5 cm | 5.7 cm | 2.3 cm | 2.4 cm | 3.1 cm |
|  | 5 cm | 4 cm | 4 cm |  | 2.8 cm | 2.9 cm | 2.1 cm |  |  | 5 cm | 5.3 cm | 4 cm | 3.1cm | 2.7 cm | 2.8 cm | 5.3 cm |
|  | 2.5 cm | 3.5 cm | 4 cm |  | 3 cm | 2.7 cm | 2.5 cm |  |  | 4.5 cm | 4.3 cm |  | 6 cm | 2.8 cm | 2.6 cm | 2.3 cm |
|  | 6 cm | 3.3 cm | 5 cm |  | 2.5 cm | 2.7 cm | 2.8 cm |  |  | 2 cm | 4.8 cm |  | 6 cm | 2.3 cm | 2.4 cm | 2.6 cm |
|  | 3.5 cm |  | 4.2 cm |  | 2.8 cm | 2 cm | 2.1 cm |  |  | 5 cm | 4.7 cm |  | 2.5 cm | 2.4 cm | 2 cm | 2.6 cm |
|  | 2 cm |  | 5 cm |  | 3.2 cm | 3.1 cm | 2.5 cm |  |  | 4.5 cm | 5.1 cm |  | 2.5 cm | 2.6 cm | 2.7 cm | 2.4 cm |
|  | 3.2 cm |  | 3.5 cm |  | 3 cm | 3 cm | 5 cm |  |  |  | 4.9 cm |  | 5.2 cm | 2.1 cm | 2.3 cm |  |
|  | 2 cm |  |  |  | 3 cm | 2.7 cm | 2.8 cm |  |  |  |  |  | 2.4 cm | 2.2 cm | 2.6 cm |  |
|  | 4 cm |  |  |  | 2.5 cm | 2.6 cm | 5.5 cm |  |  |  |  |  | 3.3 cm | 2.5 cm | 2.2 cm |  |
|  | 2.5 cm |  |  |  | 3 cm | 3 cm | 2.6 cm |  |  |  |  |  | 2.8 cm | 2.2 cm | 2.4 cm |  |
|  | 4.5 cm |  |  |  |  | 2.3 cm | 2.5 cm |  |  |  |  |  | 6 cm | 2.5 cm | 2.9 cm |  |
|  | 2.5 cm |  |  |  |  |  | 2.1 cm |  |  |  |  |  | 3 cm | 2.2 cm | 2.5 cm |  |
|  | 3 cm |  |  |  |  |  | 2 cm |  |  |  |  |  | 2.3 cm | 2.8 cm | 2.4 cm |  |
|  | 3 cm |  |  |  |  |  | 2.6 cm |  |  |  |  |  | 2.7 cm |  | 2.8 cm |  |
|  |  |  |  |  |  |  | 2.7 cm |  |  |  |  |  | 2.7 cm |  |  |  |
|  |  |  |  |  |  |  |  |  |  |  |  |  | 2.6 cm |  |  |  |
|  |  |  |  |  |  |  |  |  |  |  |  |  | 2.8 cm |  |  |  |
|  |  |  |  |  |  |  |  |  |  |  |  |  | 2.4 cm |  |  |  |
| Adults |  |  |  |  |  |  |  |  |  |  |  |  |  |  |  |  |
| Subadults |  |  |  |  |  |  |  |  |  |  |  |  |  |  |  |  |
| Juveniles |  |  |  |  |  |  |  |  |  |  |  |  |  |  |  |  |
