# Supplementary material for: Ranavirus genotypes in the Netherlands and their potential association with virulence in water frogs (Pelophylax spp.)
Source: Emerg Microbes Infect. 2018 Apr 4;7:56. doi: 10.1038/s41426-018-0058-5 (PMC5882854; doi:10.1038/s41426-018-0058-5)
Supplement: Supplementary file 9 — Table S4(DOCX 13 kb) [file 41426_2018_58_MOESM9_ESM.docx]

**Supplementary Table S4** General isolate data

| **Isolate no.** | **Isolate name** | **Province/year** | **Genbank no.** | **Species** | **Coordinates** |
| --- | --- | --- | --- | --- | --- |
| 1 | UU3110504006 | Overijssel/2011 | MF004271 | *Pelophylax spp* | 6.31, 52.65 |
| 2 | UU3110920007 | Drenthe/2011 | MF038789 | *Pelophylax spp* | 6.47, 52.75 |
| 3 | UU3110810001 | Drenthe/2011 | MF033604 | *Pelophylax spp* | 6.21, 52.77 |
| 4a | UU3120627007 | Overijssel/2012 | MF062693 | *Pelobates fuscus* | 6.28, 52.63 |
| 4b | UU3150625026 | Overijssel/2015 | MF102029 | *Lissotriton vulgaris* | 6.28, 52.63 |
| 5 | UU3140911033 | Drenthe/2014 | MF102028 | *Pelophylax spp* | 6.37, 52.73 |
| 6a | UU3130829033 | Friesland/2013 | MF062694 | *Lissotriton vulgaris* | 6.29, 52.94 |
| 6b | UU3140624035 | Friesland/2014 | MF062695 | *Pelophylax spp* | 6.29, 52.94 |
| 7a | UU3140708068 | Friesland/2014 | MF093732 | *Pelophylax spp* | 6.38, 52.97 |
| 7b | UU3150902051 | Friesland/2015 | MF004272 | *Pelophylax spp* | 6.38, 52.97 |
| 8 | UU3150902003 | Drenthe/2015 | MF102030 | *Pelophylax spp* | 6.50, 52.98 |
| 9 | UU3151009019 | Gelderland/2015 | MF125269 | *Pelophylax spp* | 6.28, 51.98 |
| 10 | UU3160714042 | Limburg/2016 | MF125270 | *Pelophylax spp* | 6.04, 51.16 |
