# Supplementary material for: Ranavirus genotypes in the Netherlands and their potential association with virulence in water frogs (Pelophylax spp.)
Source: Emerg Microbes Infect. 2018 Apr 4;7:56. doi: 10.1038/s41426-018-0058-5 (PMC5882854; doi:10.1038/s41426-018-0058-5)
Supplement: Supplementary file 10 — Supplementary figure legends(DOCX 14 kb) [file 41426_2018_58_MOESM10_ESM.docx]

**Supplementary Figure S1.** Mutations among CMTV-NL group I. Overall mutations among CMTV-NL group I ranged from 5-31, accounting for roughly 0.02% mutations in the genome (107 kbp). There is no clear pattern or correlation among distance to the site closest of the original outbreak and number of mutations.

**Supplementary Figure S2:** Statistical model details

**Supplementary Figure S3.** Map of viral isolates for which site numbers can be correlated with general viral isolate data available in Supplementary table

**Supplementary Figure S4.** Monitored sites in National Park Dwingelderveld (Top panel) and De Driestruik (Bottom panel). Studied water bodies are indicated by black squares. From all studied sites only DNP-III is a fen, the rest are ponds. The distance between ponds ranges from 300-800 meters. Only the site DD-I had no previous history of ranavirus presence. XY Coordinates given in decimals are as follows: DD-I, 51.166536, 6.028247, DD-II 51.165470, 6.035933 DD-III, 51.166383,6.028254 DNP-I, 52.7815,6.3730, DNP-II 52.7819,6.3739, DNP-III, 52.7899,6.3701. Color scheme: Waterbodies in blue, heathlands in pink, buildings in gray, forest areas in green, sand areas in yellow.
